# Assessing Health Technology Literacy and Attitudes of Patients in an Urban Outpatient Psychiatry Clinic: Cross-Sectional Survey Study

**DOI:** 10.2196/63034

**Published:** 2024-12-30

**Authors:** Julia Tartaglia, Brendan Jaghab, Mohamed Ismail, Katrin Hänsel, Anna Van Meter, Michael Kirschenbaum, Michael Sobolev, John M Kane, Sunny X Tang

**Affiliations:** 1 Department of Psychiatry Northwell Health Zucker Hillside Hospital Glen Oaks, NY United States; 2 Department of Psychiatry Weill Cornell Medical College New York, NY United States; 3 Department of Child and Adolescent Psychiatry School of Medicine NYU Grossman New York, NY United States; 4 Cornell Tech Cornell University New York, NY United States

**Keywords:** digital literacy, attitudes, mental health, digital health technology, cluster analysis, psychiatry, mobile phone

## Abstract

**Background:**

Digital health technologies are increasingly being integrated into mental health care. However, the adoption of these technologies can be influenced by patients’ digital literacy and attitudes, which may vary based on sociodemographic factors. This variability necessitates a better understanding of patient digital literacy and attitudes to prevent a digital divide, which can worsen existing health care disparities.

**Objective:**

This study aimed to assess digital literacy and attitudes toward digital health technologies among a diverse psychiatric outpatient population. In addition, the study sought to identify clusters of patients based on their digital literacy and attitudes, and to compare sociodemographic characteristics among these clusters.

**Methods:**

A survey was distributed to adult psychiatric patients with various diagnoses in an urban outpatient psychiatry program. The survey included a demographic questionnaire, a digital literacy questionnaire, and a digital health attitudes questionnaire. Multiple linear regression analyses were used to identify predictors of digital literacy and attitudes. Cluster analysis was performed to categorize patients based on their responses. Pairwise comparisons and one-way ANOVA were conducted to analyze differences between clusters.

**Results:**

A total of 256 patients were included in the analysis. The mean age of participants was 32 (SD 12.6, range 16-70) years. The sample was racially and ethnically diverse: White (100/256, 38.9%), Black (39/256, 15.2%), Latinx (44/256, 17.2%), Asian (59/256, 23%), and other races and ethnicities (15/256, 5.7%). Digital literacy was high for technologies such as smartphones, videoconferencing, and social media (items with >75%, 193/256 of participants reporting at least some use) but lower for health apps, mental health apps, wearables, and virtual reality (items with <42%, 108/256 reporting at least some use). Attitudes toward using technology in clinical care were generally positive (9 out of 10 items received >75% positive score), particularly for communication with providers and health data sharing. Older age (*P*<.001) and lower educational attainment (*P*<.001) negatively predicted digital literacy scores, but no demographic variables predicted attitude scores. Cluster analysis identified 3 patient groups. Relative to the other clusters, cluster 1 (n=30) had lower digital literacy and intermediate acceptance of digital technology. Cluster 2 (n=50) had higher literacy and lower acceptance. Cluster 3 (n=176) displayed both higher literacy and acceptance. Significant between-cluster differences were observed in mean age and education level between clusters (*P*<.001), with cluster 1 participants being older and having lower levels of formal education.

**Conclusions:**

High digital literacy and acceptance of digital technologies were observed among our patients, indicating a generally positive outlook for digital health clinics. Our results also found that patients of older age and lower formal levels of educational attainment had lower digital literacy, highlighting the need for targeted interventions to support those who may struggle with adopting digital health tools.

## Introduction

### The Digital Psychiatry Revolution

The health care system is undergoing a digital evolution, driven by rapid technological advancements. “Digital health” is defined as “the use of information and communications technologies in medicine to manage illnesses and health risks and to promote wellness” [1]. Over the past decade, digital health has been met with enthusiasm from both patients and providers due to its potential to revolutionize the delivery of mental health services, making mental health treatment more affordable and accessible [2-4]. The mental health sector has been an early adopter of this technological integration, a trend accelerated by the COVID-19 pandemic. During this period, there has been a significant increase in the use of digital mental health technologies, such as telehealth services and mental health apps. A recent review and meta-analysis found that these digital interventions were associated with reductions in depressive and anxiety symptoms, underscoring the importance of these tools during the pandemic [5]. The increased reliance on digital tools has expanded access and highlighted the need to understand patients’ current attitudes and literacy levels. As the pandemic necessitated remote care, both patients and providers have become more accustomed to digital health technologies, potentially shifting the landscape of mental health care delivery.

Digital psychiatry is a broad term that extends beyond telehealth, encompassing patient/provider information and communication tools (eg, electronic medical records, patient portals, and telepsychiatry videoconferencing platforms), digital interventions (eg, therapy delivered through text messaging, smartphone apps, virtual reality [VR] headsets, or video games), symptom-monitoring tools (eg, self-report symptom-tracking or health data collected through wearable devices), and predictive machine learning tools that seek to use passive data from smartphones and wearable devices to potentially predict major mood episodes and psychotic relapses, offering a glimpse into the future capabilities of digital health [6-8].

As digital approaches become more common, interest has grown in creating digital mental health clinics—new care delivery models that integrate digital tools into clinical practice [9-11]. Such clinics can potentially ameliorate existing disparities in health care accessibility and promote health equity among psychiatric patient populations.

### Health Equity and the Digital Divide

Despite the benefits offered by digital psychiatry tools, an over-reliance on digital tools could inadvertently exacerbate pre-existing disparities in health care access between those who can use the technology and those who cannot. This is a concept known as the digital divide [12]. A variety of documented factors contribute to this inequity in the use of health technology, including differences in access (ie, home Wi-Fi/broadband connectivity), proficiency or technological skills required to engage with these tools effectively, and attitudes toward the use of technology in mental health care [12-14]. For this paper, we group accessibility and usability broadly under the term “digital literacy,” which United Nations Educational, Scientific and Cultural Organization (UNESCO) defines as “the ability to access, manage, understand, integrate, communicate, evaluate and create information safely and appropriately through digital technologies” [15]. Within a health care context, digital literacy pertains to an individual’s ability to access digital tools, to understand health information from electronic sources, and to successfully use digital tools—including patient portals, videoconferencing, smartphone apps, wearable devices, and health trackers—both as interventions and for communication purposes [14].

### Digital Access and Literacy

The existing literature highlights notable disparities in digital access that correlate with demographic variables, such as age, race, ethnicity, income, and educational attainment. While a high percentage of US adults own smartphones, access is not uniform across all groups. Ownership rates are marginally lower among individuals from low-income households, Black and older adults, and non–college-educated individuals [16]. Moreover, the same groups tend to be affected by the lack of home broadband connectivity among 15% of smartphone owners, further complicating their engagement with digital health services. Broadband access, in particular, is a critical issue, as an estimated 21 million people in the United States lack adequate broadband connectivity. This digital divide disproportionately affects underserved populations. Including rural residents and racial and ethnic minorities, limiting their ability to benefit from digital health advancements [17]. This uneven access underscores the need for targeted interventions that address the specific barriers these populations face in using digital health tools effectively.

Beyond access issues, differences in the techniques and skills, and abilities required to use digital health tools successfully have been found in certain demographic groups. In particular, older age has been linked to lower digital literacy. A UK study examining digital technology use among psychiatric patients found that older adults reported less familiarity and confidence using various mobile and computer devices [18]. Furthermore, a US study of adults aged 50 years and older found that while usage rates of email, SMS text messaging, and health applications were similar across racial and ethnic groups, older Black and Hispanic individuals were less likely to use patient portals and search online for health information compared with White older adults [19]. Language barriers also present significant challenges, as studies have found that a majority of health apps are monolingual, operating solely in English, thereby limiting use by non–English speakers [20].

### Digital Acceptability

Finally, differences in an individual’s interest and motivation to use technology for mental health care can impact adoption independent of one’s ability. Personal beliefs about technology have been found to impact engagement with digital health tools—patients who are uninterested or have negative attitudes toward digital health tools could become “self-excluders,” thus exacerbating the risk of digital exclusion [18].

Furthermore, identifying patients who have positive attitudes toward technology is equally important. For example, a study of digital technology use in older adult patients found that while they reported literacy-related barriers to adoption, they had favorable attitudes toward using digital mental health tools [21]. Identifying patients with high digital acceptability but lower literacy levels can help clinics target specific patients with resources, such as digital navigator support [22]. Currently, little is known about the general attitudes of patients toward mental health technology use in outpatient clinics or the patient characteristics that impact digital acceptability.

### No Patient Left Offline: Bridging a Digital Divide

To successfully develop a digital psychiatric clinic without widening health care disparities, it is crucial to address the factors that contribute to a digital divide. Clinic leadership must work to identify patient groups that might be marginalized by the shift to digital workflows, screening, and interventions so that targeted measures, such as enhanced technical support, resources for digital navigation, education about the benefits of digital tools, or even opting to retain traditional methods can be deployed, as appropriate.

Moreover, comprehending the interplay between digital literacy and the willingness to use digital tools is essential. It ensures that these innovations reach those who are open and stand to benefit from them, thereby optimizing patient engagement and outcomes.

### Objectives

Our objective was to identify clusters of patients based on patterns in their expressed digital health literacy and attitudes and to compare sociodemographic characteristics among the clusters. To accomplish this, we elicited patient responses to survey questions during intake for an outpatient treatment program in an urban, racially, and socioeconomically diverse area.

Our research questions were as follows:

1. What are the current states of digital literacy and attitudes toward digital health in this urban, racially, and socioeconomically diverse outpatient psychiatric population?
2. Do patient characteristics such as race and ethnicity, age, gender, level of education, and marital status influence digital attitudes or digital literacy?
3. Are there patterns or subgroups among our patients with regard to digital literacy and attitudes? If so, do they differ by patient characteristics?

## Methods

### Study Design

This study used an institutional review board–approved retrospective chart review design to examine the patient characteristics, digital literacy, and attitude outcomes of patients at an urban adult psychiatric outpatient clinic. During a 2-year period, a voluntary electronic health care technology survey was administered via REDCap (Research Electronic Data Capture, Vanderbilt University) to all new patient intakes at 4 adult ambulatory psychiatry clinics as part of routine intake paperwork. The survey was administered as part of an initial pilot project to develop a psychiatric digital health program. The response rate was around 40%. Due to the practical constraints of recruiting patients during routine intake in a clinical setting, a nonprobability sampling method was used. While this approach may limit the generalizability of the findings to broader populations, it allows for an in-depth exploration of digital literacy and attitudes in this specific urban outpatient psychiatry content.

### Ethical Considerations

All study procedures were reviewed and approved by the Feinstein Institutes for Medical Research IRB (#23-0667-ZHH). As the research was conducted using deidentified data previously collected for clinical and quality purposes, informed consent was waived.

### Study Setting

The outpatient program, located in Queens, New York, consists of multiple clinics, including the Adult Outpatient Psychiatry Department, the Bipolar Disorder Clinic, the Behavioral Health College Partnership program, and the Early Treatment Program for first-break psychosis. The clinics offer comprehensive mental health outpatient services for adults with psychiatric conditions, including mood disorders, anxiety disorders, psychotic spectrum illnesses, and substance use issues. The patient population generally consists of patients who reside in Long Island, Queens, and Brooklyn, and as such, reflects the diversity of the surrounding community, which varies greatly by race and ethnicity, socioeconomic status, age, gender identity and orientation, education, and family structure.

### Measures and Questionnaires

The survey contained 3 self-assessment questionnaires of interest to our team: a demographic questionnaire, a digital literacy survey, and a digital health attitudes questionnaire. We made the questionnaires publicly available on the Open Science Framework repository [23]. The complete questionnaires can also be viewed in Multimedia Appendix 1.

### Demographic Data and Patient Characteristics

The patient’s demographic characteristics, including race and ethnicity, education level, employment status, age, sex, gender, and marital status, were ascertained through a self-reported questionnaire. Income level was not included as part of the survey; thus, employment status and education are used as a rough proxy for economic status.

### Digital Literacy Scale

The participants were given a 10-item “Digital Literacy” scale created by the team developing the psychiatric digital health program. The scale reported good internal consistency (Cronbach α=0.85). The participants were asked to rank their familiarity and frequency of use of several different technologies. The items included online shopping, web searches, social media, videoconferencing, smart speakers, smartphones, wearable devices, health tracking apps, mental health apps, and VR. Items were scored on a 4-point Likert scale, with 1 being no familiarity with and no use of the technology and 4 being the highest familiarity and use of the technology. A total digital literacy score was generated for each participant by calculating the unweighted sum of the 10 individual items. The complete questionnaire is provided in the Multimedia Appendix 1.

### Digital Attitudes Scale

The participants were also surveyed on their attitude toward using digital care for mental health using a 10-item questionnaire (or “Attitudes” scale) created by the team developing the psychiatric digital health program. The scale reported good internal consistency (Cronhbach α=0.85). The questions address participants’ willingness to use different technologies for aspects of their mental health care. The participants were asked to rank each question on a 5-point Likert scale ranging from strongly disagree (score of 1) to strongly agree (score of 5). The items assessed attitudes toward three types of tools: (1) self-help and self-monitoring tools (eg, “Tracking my own symptoms (eg, mood, anxiety) using a web or mobile app can support my mental health”), (2) tools that assist in communication with providers (“eg, ability to communicate with my care team via text messages or mobile app can improve my care”), and (4) tools that share health data with providers (eg, “Automatically sharing info about my daily activities [eg, sleep and physical activity] with my care team can improve my mental health” or “Sharing information about my online activity [eg, Google searches and Facebook posts] can improve my care]”). A total Attitudes score was generated for each participant by taking the unweighted sum across all 10 items. The complete questionnaire is provided in Multimedia Appendix 1.

### Data Extraction and Preparation

Our inclusion criteria consisted of all patients who completed the health care technology survey (Attitude and Literacy scales) on intake. The survey was active between November 21, 2020 and January 17, 2022. A total of 286 charts were extracted. Test entries (n=2) and charts with incomplete Digital Literacy or Attitudes scales (n=28) were removed from the dataset. A total of 256 participants were included in the final analysis. For the remaining cases, missing values in the demographics scale were indicated as “not reported” and excluded from any relevant analyses.

### Statistical Analyses

All data analyses were conducted using R (version 4.2.2; R Development Core Team).

#### Linear Regressions

Multiple linear regression analyses evaluated the relationships between patient characteristics (independent variables) and the total Digital Literacy and Attitudes scores (dependent variables). This approach was chosen to allow for simultaneous evaluation of multiple predictors while controlling for covariates. Standardized β coefficients were reported. Before conducting the regressions, we tested the assumptions of normality and homoscedasticity of residuals. The normality of the dependent variable (Digital Literacy and Attitudes scores) was assessed using the Shapiro-Wilk test, residual plots were visually inspected for homoscedasticity, and the data were determined to satisfy normality assumptions. Statistical significance was determined using a 2-sided α level of .05.

#### Cluster Analysis

A 2-step cluster analysis approach was performed to identify subgroups within the patient sample based on their responses to the literacy and attitude scales. First, we used the *NbClust* in R to determine the optimal number of clusters. A Euclidean distance matrix was calculated to measure dissimilarities between participants. K-means clustering was then performed to produce 3 clusters, iteratively assigning each participant to one of the clusters to minimize the within-cluster sum of squares.

#### Pairwise Comparisons

To compare the differences in patient characteristics across cluster groups, group comparisons were conducted using either *t* test for continuous variables or chi-square test for categorical variables. For continuous variables (age, Literacy score, and Attitudes score), significance was tested using ANOVA. For categorical variables (race, education, gender, gender, employment, sex, and marital status), significance was tested with chi-square test. For variables with significant differences across clusters, pairwise comparisons using *t* tests with pooled SD were conducted to assess the differences in patient characteristics between clusters. The *P* values were adjusted using the false discovery rate (FDR) correction method [24].

## Results

### Patient Characteristics

The participant characteristics are summarized in Table 1. The mean age was 32 (SD 12.6, range 16-70) years. Notably, the patient population was diverse and representative of the New York City metropolitan area: White (100/256, 38.9%), Black (39/256, 15.2%), Latinx (44/256, 17.2%), Asian (59/256, 23%), and other races and ethnicities (15/256, 5.7%). Most patients (179/256, 70.1%) were single. Self-reported gender and sex were roughly equal between males and females. The majority (196/254, 77.2%) of the patients obtained some level of postsecondary education. There was a distribution in education status across those who were retired/on disability, unemployed, employed, or student. Socioeconomic status was inferred from data on employment status and educational attainment rather than directly solicited from respondents.

**Table 1:** Participant characteristics by total and by cluster.

Characteristics		Cluster 1 (n=30)	Cluster 2 (n=50)	Cluster 3 (n=176)	Total (N=256)	*P* value^a^	
**Age**							*<.001* ^b^
	Mean (SD)	40.5 (16.3)	33.9 (12.6)	30.3 (11.2)	32.3 (12.6)		
	Range	20.0-66.0	19.0-63.0	16.0-70.0	16.0-70.0		
	Not reported, n	0	0	4	4		
**Gender**							.32
	Female, n (%)	12 (42.9)	26 (54.2)	100 (56.8)	138 (54.8)		
	Male, n (%)	16 (57.1)	22 (45.8)	71 (40.3)	109 (43.3)		
	Other^c^, n (%)	0 (0)	0 (0)	5 (2.8)	5 (2)		
	Not reported, n	2	2	0	4		
**Race and ethnicity**							.32
	White, n (%)	13 (50)	20 (42.6)	62 (36.3)	95 (38.9)		
	Black, n (%)	2 (7.7)	7 (14.9)	28 (16.4)	37 (15.2)		
	Latinx^d^, n (%)	5 (19.2)	12 (25.5)	25 (14.6)	42 (17.2)		
	Asian, n (%)	4 (15.4)	6 (12.8)	46 (26.9)	56 (23)		
	Other, n (%)	2 (7.7)	2 (4.3)	10 (5.8)	14 (5.7)		
	Not reported, n	4	3	5	12		
**Education**							*<.001*
	Some high school, n (%)	9 (30)	2 (4.1)	10 (5.7)	21 (8.3)		
	Completed high school^e^, n (%)	6 (20)	4 (8.2)	27 (15.4)	37 (14.6)		
	Some college^f^, n (%)	10 (33.3)	26 (53.1)	70 (40)	106 (41.7)		
	College, n (%)	3 (10)	10 (20.4)	33 (18.9)	46 (18.1)		
	Graduate school^g^, n (%)	2 (6.7)	7 (14.3)	35 (20)	44 (17.3)		
	Not reported, n	0	1	1	2		
**Employment status**							.06
	Retired, n (%)	1 (3.3)	2 (4.1)	3 (1.7)	6 (2.4)		
	Disability, n (%)	5 (16.7)	1 (2)	14 (8)	20 (7.8)		
	Not employed^h^, n (%)	11 (36.7)	22 (44.9)	48 (27.3)	81 (31.8)		
	Employed part-time, n (%)	6 (20)	5 (10.2)	22 (12.5)	33 (12.9)		
	Employed full-time, n (%)	4 (13.3)	12 (24.5)	43 (24.4)	59 (23.1)		
	Student, n (%)	3 (10)	7 (14.3)	46 (26.1)	56 (22)		
	Not reported, n	0	1	0	1		
**Marital status**							.19
	Single, n (%)	17 (58.6)	31 (66)	128 (73.1)	176 (70.1)		
	Divorced or widowed, n (%)	2 (6.9)	5 (10.6)	6 (3.4)	13 (5.2)		
	Married, n (%)	10 (34.5)	11 (23.4)	41 (23.4)	62 (24.7)		
	Not reported, n	1	3	1	5		

^a^*P* value shown for group comparisons using either a *t* test for continuous variables or a chi-square test for categorical variables.

^b^*P* values less than .005 are considered statistically significant and are italicized.

^c^Other includes participants who indicated “Other” or whose race or ethnicity was not reported.

^d^“Latinx” includes all participants who indicated their ethnicity as Latino or Hispanic, including Black Hispanic or Latino and White Hispanic or Latino.

^e^“Completed high school” includes high school and General Educational Development Test.

^f^“Some College” includes completed two-year college or technical program.

^g^“Graduate School” includes completed graduate degree or some graduate school.

^h^“Not employed” includes unemployed and retired.

### Digital Literacy and Attitudes Scale Results

**Figure 1:**
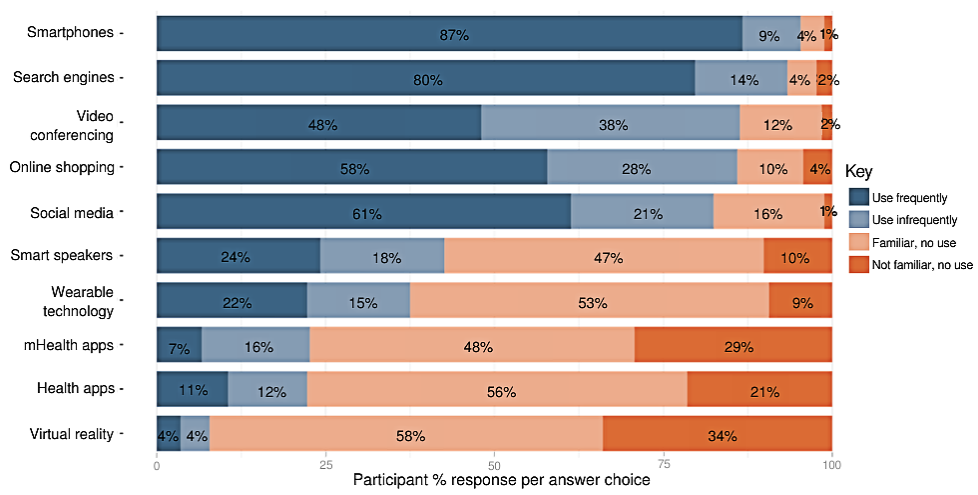
Digital Literacy Scale response percentage by individual item. The distribution of technological literacy among study participants, segmented by their frequency of technology use and familiarity. Each category represents the percentage of participants (N=256) who regularly use, occasionally use, are familiar with but do not use, or are not familiar with various digital technologies. Data are presented in descending order based on the combined percentages of participants who either use the technology frequently or infrequently.

The findings from the Digital Literacy and Attitudes scales are displayed in Figure 1, which shows the total responses for each item. Overall, the participants reported a fair level of digital literacy, as indicated by high levels of familiarity and use of digital tools (Figure 1). For 5 out of the 10 items (social media, videoconferencing, web-based shopping, search, and smartphone use), over 75% of participants reported at least some use of the technology. Patients’ most frequently used technologies were smartphones, search engines, videoconferencing, web-based shopping, and social media, respectively. Patients reported the least familiarity and use of VR tools, health apps, mental health apps, wearable technology, and smart speakers.

**Figure 2:**
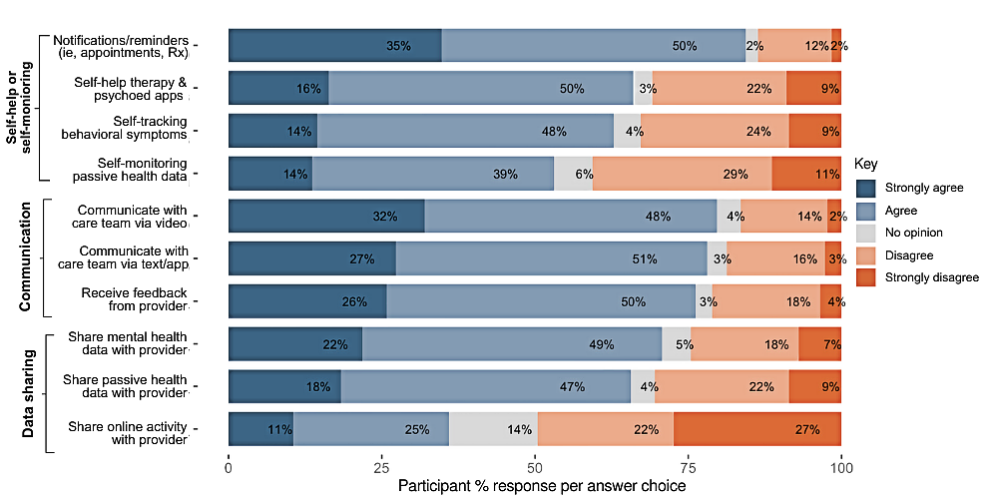
Digital Attitudes Scale response percentage by individual item. The percentage distribution of responses across various digital health communication preferences (N=256). Responses were grouped and ordered based on predefined categories reflecting different aspects of digital health communication.

The participants also demonstrated positive attitudes toward using technology in their mental health care, with over 50% favorable responses (agree or strongly agree) in 9 out of the 10 items (Figure 2). Notifications, reminders, and communication with the treatment team were the most acceptable uses of technology, followed by self-help and self-monitoring. Sharing online activity with providers (eg, social media and search activity) was the least favorable, with 49% (125/256) of participants disagreeing that this would be helpful to their care. Attitudes toward self-monitoring one’s own passive data were similarly divided, with 53% (136/256) reporting positive attitudes and 40% (102/256) reporting negative attitudes.

### Relationship Between Patient Characteristics and Digital Literacy and Attitudes Total Scores

Multiple linear regression analyses were conducted to determine the relationship between patient characteristics, Digital Literacy, and Attitudes scale scores. The results are summarized in Tables 2 and 3. Age was negatively associated with digital literacy (*P*<.001). Higher educational attainment was associated with higher literacy total scores (*P*<.03 to *P*<.001).

There were no statistically significant associations between patient characteristics and attitude scores. When adding the total Literacy score into the model, Literacy total was found to positively correlate with Attitudes total scores (estimate 0.33, SE 0.12, t_214_=2.68; *P*=.001).

In Table 2, the literacy model for total scores yielded a residual SE of 4.373 on a df of 214, with 23 observations omitted for missingness (multiple *R*^2^=0.209; adjusted *R*^2^=0.1423; *F*_18,214_=3.141; *P*=3.747×10^–5^). Table 2 illustrates the coefficients from multiple linear regression analyses assessing the impact of patient demographics on literacy total scores. Coefficients represent the estimated change in the total score for a one-unit increase in the predictor variable, holding other variables constant. SEs, *t* values, and associated *P* values are provided for each coefficient. The reference category for each categorical variable is denoted by a coefficient of 1.00.

Table 3 illustrates the coefficients from multiple linear regression analyses assessing the impact of patient demographics on attitude total scores. Coefficients represent the estimated change in the total score for a one-unit increase in the predictor variable, holding other variables constant. SEs, *t* values, and associated *P* values are provided for each coefficient. The reference category for each categorical variable is denoted by a coefficient of 1.00. The attitude model for total scores yielded a residual SE of 7.902 on a df of 214, with 23 observations omitted for missingness (multiple *R*^2^=0.05458; adjusted *R*^2^=–0.0249; *F*_18,214_=0.6863; *P*=.82). Observations with missing values were excluded from the analysis.

**Table 2:** Multiple linear regression analysis–impact of patient characteristics on Literacy Total Score predicted by patient characteristics.

Variable		Coefficient (SE)	*t* value (*df*)	*P*_r(>|t|)_	Significance
(Intercept)		31.73 (3.24)	9.80 (214)	<.001	<.001
Age		–0.11 (0.03)	–3.5 (214)	<.001	<.001
**Gender**					
	Female	1 (reference)	—^a^	—	—
	Male	–0.91 (0.63)	–1.44 (214)	.15	—
	Other	2.14 (2.31)	0.93 (214)	.36	—
**Race**					
	White	1 (refrence)	—	—	—
	Black	–0.15 (0.94)	–0.17 (214)	.87	—
	Latinx	0.05 (0.89)	0.06 (214)	.96	—
	Asian	–0.53 (0.80)	–0.66 (214)	.51	—
	Other	–3.46 (1.30)	–2.66 (214)	*.008* ^b^	<.01
**Education**					
	Some high school	1 (reference)	—	—	—
	High school	2.43 (1.31)	1.85 (214)	*.07*	<.1
	Some college	2.5 (1.16)	2.16 (214)	*.03*	<.05
	College	2.6 (1.3)	2.00 (214)	*.05*	<.05
	Graduate school	4.46 (1.29)	3.47 (214)	*<.001*	<.001
**Employment**					
	Not employed	1 (reference)	—	—	—
	Disability	–3.08 (2.38)	–1.24 (214)	.20	—
	Unemployed	–2.34 (2.28)	–1.03 (214)	.31	—
	Employed part-time	–1.98 (2.37)	–0.83 (214)	.41	—
	Student	–0.91 (2.42)	–0.38 (214)	.71	—
	Employed	–0.64 (2.22)	–0.29 (214)	.78	—
**Marital Status**					
	Divorced/widowed	1 (reference)	—	—	—
	Single	0.12 (1.44)	0.09 (214)	.93	—
	Married	0.73 (1.48)	0.49 (214)	.63	—

^a^Not applicable.

^b^*P* values less than .005 are considered statistically significant and are italicized.

**Table 3:** multiple linear regression analysis–impact of patient characteristics on Attitude total score predicted by patient characteristics.

Variable			Coefficient (SE)		*t* value (*df*)					*P*_r(>|t|)_	Significance	
(Intercept)			33.38 (5.85)		5.71 (214)					<.001	<.001	
Age			–0.04 (0.06)		–0.62 (214)					.54	—^a^	
**Gender**												
	Female	1 (reference)					—		—			—
	Male	0.82 (1.14)					–0.72 (214)		.47			—
	Other	1.14 (4.17)					0.27 (214)		.78			—
**Race**												
	White	1 (reference)					—		—			—
	Black	0.32 (1.69)					0.19 (214)		.85			—
	Latinx	2 (1.61)					1.25 (214)		.21			—
	Asian	1.13 (1.45)					0.78 (214)		.44			—
	Other	1.47 (2.35)					0.63 (214)		.53			—
**Education**												
	Some high school	1 (reference)					—		—			—
	High school	3.62 (2.37)					1.52 (214)		.13			—
	Some college	1.97 (2.09)					0.95 (214)		.35			—
	College	1.76 (2.34)					0.75 (214)		.45			—
	Graduate school	3.31 (2.32)					1.43 (214)		.16			—
**Employment**												
	Not employed			1 (reference)		—		—			—	
	Disability			–1.72 (4.30)		–0.40 (214)		.69			—	
	Unemployed			–0.92 (4.12)		–0.22 (214)		.82			—	
	Employed part-time			0.93 (4.29)		0.22 (214)		.83			—	
	Student		1.15 (4.38)			0.26 (214)		.80			—	
	Employed		1.27 (4.02)			0.32 (214)		.75			—	
**Marital status**												
	Divorced/widowed		1 (reference)			—		—			—	
	Single		0.58 (2.60)			0.22 (214)		.82			—	
	Married		–0.12 (2.67)			–0.05 (214)		.96			—	

^a^Not applicable.

### Cluster Analysis Findings

A cluster analysis was conducted to group participants based on similarities in their responses to the Attitude and Literacy scales using the previously described method. A total of 3 distinct participant clusters were identified (Table 1). Cluster 1 had 30 participants, cluster 2 had 50 participants, and cluster 3 had 176 participants.

Using pairwise comparisons conducted using *t* tests and pooled SD, we examined the relative differences in Literacy total scores between the participant clusters (Figure 2). There was a significant difference in Literacy total scores between cluster 1 and the other two clusters (*P*<.001 vs cluster 2, and *P*<.001 vs cluster 3), indicating that cluster 1 had relatively lower total literacy scores than clusters 2 and 3. The median item-level score was 2, “Familiar but no use.” There was no significant difference in Literacy total scores between clusters 2 and 3 (*P*=.07). Median item-level scores were 3, and 3, respectively, corresponding to “Use infrequently.”

The results of the pairwise comparisons of the Attitude total scores across the different clusters revealed significant differences in attitudes among all three clusters. Cluster 1 Attitude total scores were significantly higher than cluster 2 (*P*<.001) and lower than cluster 3 (*P*<.001), with a median item-level score of 3, corresponding to “No opinion.” Cluster 2 Attitude total was significantly lower than both clusters 1 and 3 (*P*<.001), with a median item-level score of 2, corresponding to “disagree.” Cluster 3 Attitude scores were significantly higher than both clusters 1 and 2 (*P*<.001), with a median item-level score of 4, corresponding to “Agree.” After adjusting for multiple comparisons using the FDR method, the results demonstrated consistent findings, reinforcing the significant differences in attitudes across all pairs of clusters.

**Figure 3:**
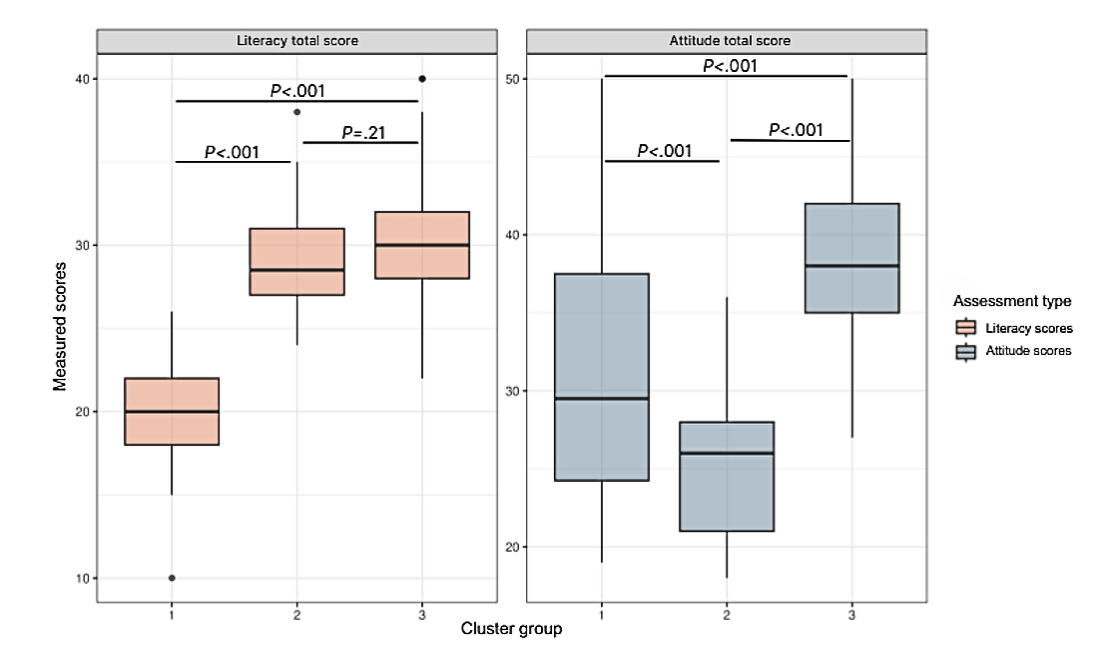
Cluster analysis–comparative distribution of Attitude and Literacy scores across clusters. Box plots illustrate the total scores for Attitude and Literacy compared across the three clusters. Pairwise comparisons were conducted using t tests with pooled SD. An initial one-way ANOVA was performed for each variable, indicating significant differences among the clusters for both Attitude (F[2-253]=99.16; *P*<.001) and Literacy (F>2-253]=112.6; *P*<.001). Subsequent pairwise t tests corrected for multiple comparisons using the Benjamini-Hochberg method identified specific clusters between which significant differences exist; these are denoted on the plots with horizontal lines and labeled with exact *P* values.

Taken together, the clusters were characterized based on their relative Attitude total and Literacy total scores. Cluster 1 demonstrated lower literacy levels and intermediate acceptance of digital technology relative to the other two clusters. Cluster 2 exhibited higher literacy levels and lower acceptance. Cluster 3 displayed both high literacy and acceptance (Figure 3).

### Cluster Differences by Patient Characteristics

Clusters were then compared based on key patient characteristics, as summarized in Table 1. There were statistically significant group differences across the 3 clusters for mean age (*P*<.001) and education level (*P*<.001). Employment status reached near significance (*P*=.06). No statistically significant differences between clusters were observed for race/ethnicity (*P*=.32), sex (*P*=.39), gender (*P*=.32), and marital status (*P*=.19).

Additional pairwise comparisons between clusters were completed for patient characteristic variables that exhibited statistically significant group differences (Table S1 in Multimedia Appendix 2). Regarding age, cluster 1 participants were significantly older than clusters 2 and 3 participants (*P*=.02, *P*=.03 after adjustment and *P*<.001, *P*<.001 after adjustment, respectively). There were no significant differences in age between clusters 2 and 3 (*P*=.07, .07 after adjustment).

Statistically significant differences in formal educational attainment were observed among clusters 1, 2, and 3. Specifically, cluster 1 had a higher ratio of participants who only completed some high school versus those who completed some high school or higher when compared with cluster 3 (*P* values range *P*=.02 to *P*<.001) and versus those who completed some college or higher when compared with cluster 2 (*P* value range *P*=.05 to *P*<.001). There were no significant differences in education level between clusters 2 and 3. After adjusting for multiple comparisons, group differences between clusters 1 and 3 remained statistically significant. Taken together, these findings indicate cluster 1 participants had less formal education than clusters 2 and 3.

Overall, the largest differences were found between clusters 1 and 3; on average, cluster 1 participants had lower levels of formal education and were older.

While employment did not reach statistically significant group differences, a comparison of data in Table 1 demonstrates a trend of a higher percentage of students and employed individuals in clusters 2 and 3 compared with cluster 1.

## Discussion

### Principal Findings

Our study captured a demographically and socioeconomically diverse psychiatric outpatient population. Overall responses to the Digital Literacy scale suggest patients have high literacy for certain types of digital technology, particularly smartphone use, search, videoconferencing, and social media. These tools are important for health care activities such as attending telemedicine appointments and communicating with providers. Furthermore, they support the potential of using digital phenotyping tools, which use data from social media use, internet search data, and cell phones to predict psychiatric episodes and mental health status. However, patients reported less familiarity with health apps, mental health apps, wearables, and virtual reality, suggesting that there may need to be more education or support should such tools be introduced into care.

Incorporating digital interventions into care has shown great promise in enhancing patient engagement. For example, a longitudinal study on the feasibility of a mobile health platform for behavioral care demonstrated moderate patient engagement and preliminary clinical improvements in a primary care setting, highlighting the importance of mobile platforms in augmenting collaborative care and offering psychoeducational resources through secure messaging [25]. These findings further emphasize the potential of digital tools to complement clinical practice and improve patient outcomes, particularly when supported by ongoing educational initiatives and accessible technology.

Overall responses to the Attitude scale demonstrated that our patients found the use of technology in clinical care to be highly acceptable, which aligns with prior literature highlighting favorable patient attitudes toward using telepsychiatry and mobile interventions in mental health care [21,26-28]. In our study, the most acceptable uses of technology in health care were for communication with one’s provider and both monitoring and sharing health data with providers. Patients were divided on the perceived usefulness of sharing their online activity with providers, suggesting that not all patients may be open to sharing this data with providers for digital phenotyping. More psychoeducation may be needed to explain the use of digital phenotyping to patients.

Our study highlighted several demographic factors associated with digital literacy in our patient population. Predictably, Digital Literacy scores were directly correlated with the level of formal education and indirectly correlated with age, which supports prior literature showing that older age groups are less familiar with using digital tools than younger age groups [18]. However, surprisingly, none of the patient characteristics we identified predicted total attitude scores. Notably, there were no significant differences in total digital Literacy or Attitude scores by race and ethnicity. This contradicts prior literature that found racial differences and supports the idea that digital interventions could help improve access to care and bridge health disparities. Attitude was, however, directly correlated with digital literacy scores, indicating that patients with higher familiarity and comfortability with digital tools have a higher likelihood of finding their use in clinical practice acceptable.

Our cluster analysis revealed that there are three primary groups or clusters of patients based on their relative levels of digital literacy and acceptability: those with lower digital literacy and intermediate acceptance (cluster 1), those with higher literacy but lower acceptance (cluster 2), and those that are high in both literacy and acceptance (cluster 3). Promisingly, cluster 3 was the largest, representing 69% (n/N) of the participants, suggesting that a majority of patients have both the literacy levels and interest necessary to embrace technology in clinical care. The participants with lower digital literacy levels and higher acceptance of digital technology were more likely to be older and have less formal educational attainment (cluster 1). In contrast, the other 2 groups (clusters 2 and 3) demonstrated higher literacy levels but varied regarding digital health acceptance. Our study did not find meaningful demographic differences between the higher literacy groups (clusters 2 and 3), suggesting that there are likely additional factors outside the patient characteristics examined in this study. This finding supports previous findings that lower educational attainment, which could correlate with certain professional or occupational status, can be correlated to lower digital literacy levels. For example, studies have found that people who are unemployed have lower internet use [18].

While age and educational status correlated positively with digital literacy levels, our negative findings that these same characteristics did not correlate with Attitude scores are promising, suggesting that diverse groups of patients are interested in using such technologies. We propose that patient characteristics should be considered when building out programming to determine the level of support needed to appropriately train and educate patients on how to use technologies. Particular attention should be paid to providing additional resources such as digital health navigators who can help older people with lower formal education levels. There remains a risk that if health systems do not incorporate extra support to those with lower digital literacy levels—whether due to lack of awareness or funding for additional resources—these groups will be digitally excluded, thus exacerbating existing health care disparities. In such cases, health systems should consider whether adopting digital tools would be beneficial over traditional means of care.

Given these findings, health care systems should take a proactive approach by regularly assessing digital literacy and attitudes before introducing new digital interventions. Our survey tools could serve as potential screening instruments to guide providers and clinics in determining whether digital tools are suitable for specific patient populations. Integrating these assessments into routine care would help providers tailor digital interventions to the needs of their patient populations, thus preventing digital exclusion. On a broader population level, it is important to continue examining the demographic factors that correlate with digital literacy and attitudes over time. No single screening tool has been widely adopted to assess digital literacy and attitudes across diverse patient populations, representing an important area for future research. Policy makers and health care organizations should prioritize developing and standardizing such screening tools, ensuring that digital interventions are accessible and equitable across populations.

### Limitations

This study focused on evaluating the demographic factors that may contribute to digital literacy and attitudes. Clinical factors such as diagnoses and illness severity may also play a role and should be explored as a future direction. One limitation of this study is the classification of race. The classification of race and ethnicity presents inherent challenges due to the intricacies of identity, cultural nuances, and intersectionality. Despite our efforts to meaningfully categorize individuals, distinctions such as Black Latino versus White Latino and individuals of mixed race pose complexities that cannot always be fully disentangled or accurately represented. While we have tried to use the most appropriate methodologies available, it is important to acknowledge that this process remains imperfect, constituting a notable limitation in our study’s scope. Another limitation of our study is that our survey was only available in English and likely did not capture non-English speakers. Language is a notable barrier to digital literacy, and additional studies should be conducted to examine the impact of native language on digital literacy and attitudes.

### Conclusions

Our study highlights the digital literacy of patients in an urban psychiatric outpatient clinic, particularly with commonly used technologies such as smartphones and videoconferencing tools. While most patients expressed positive attitudes toward integrating digital tools into their mental health care, overall familiarity with health-specific technologies like mental health apps and wearables was lower, especially among older individuals and those with lower educational attainment. As such, our findings emphasize the importance of addressing digital literacy gaps to ensure all patients benefit from emerging digital health solutions.

Health care systems must focus on providing tailored education and support to ensure digital tools are accessible to diverse patient populations. Hospitals and clinics can play a crucial role by integrating technology training programs for patients and providers, ensuring all users feel confident using these tools. Implementing digital health navigators, trained personnel who assist patients in using digital health platforms, can be particularly valuable for individuals with lower literacy levels, helping to close the digital divide and prevent exclusion from digital health care advancements. Policies prioritizing developing and deploying digital infrastructure in health care settings will be essential. As digital mental health tools become increasingly central to care, evaluating their acceptability and appropriateness within diverse cultural and social contexts will be key [29]. In addition, creating a regulatory framework that supports safe and effective use is vital, particularly in underserved communities. Also, policy makers should advocate for evaluating digital health tools using established frameworks like the APA (American Psychiatric Association) App Evaluation Model to ensure their efficacy and equity [30], avoiding exacerbating existing health care disparities.

In conclusion, adopting digital health tools in psychiatric care holds great promise, offering a brighter and more accessible future for mental health services. However, equitable implementation will require addressing both technological barriers and patient education. By proactively supporting digital literacy and creating inclusive digital health strategies, health care systems can enhance care delivery and promote broader access to mental health services.

## Appendix

Multimedia Appendix 1 Survey instruments.

Multimedia Appendix 2 Pairwise comparisons for patient characteristics that were significantly different in the cluster analysis (age and education).

## Abbreviations

APA: American Psychiatric Association
FDR: false discovery rate
REDCap: Research Electronic Data Capture
UNESCO: United Nations Educational, Scientific and Cultural Organization
VR: virtual reality
