# Supplementary material for: Assessing Health Technology Literacy and Attitudes of Patients in an Urban Outpatient Psychiatry Clinic: Cross-Sectional Survey Study
Source: JMIR Ment Health. 2024 Dec 30;11:e63034. doi: 10.2196/63034 (PMC11729776; doi:10.2196/63034)
Supplement: Multimedia Appendix 1 [file mental_v11i1e63034_app1.docx]

| **“Digital Technology Literacy Scale”** |
| --- |
| The question prompt was: “How familiar are you with the following technologies?” |
| Participants could choose from the following answer choices*:  I use this technology frequently  I use this technology but not so frequently  I am familiar with this technology but do not use it myself  I am not familiar with this technology |
| Questions:   1. Online shopping (Amazon, Seamless, etc.) 2. Web search (Google, Bing etc.) 3. Social media (Facebook, Twitter, Instagram, Reddit etc.) 4. Wearable devices and activity trackers (Apple watch, Fitbit, Oura ring etc. 5. Smartphones (iPhone, Google Pixel, Samsung Galaxy etc.) 6. Video conferencing (e.g. Skype, Zoom etc.) 7. Smart speakers (Amazon Echo (Alexa), Google Home etc.) 8. Virtual reality (Oculus, Google Daydream etc.) 9. Health tracking apps (MyFitnessPal, Sleep Cycle etc.) 10. Mental health apps (Headspace, Calm etc.) |
| Notes:  *Scoring: Answer choices were scored as follows:  I use this technology frequently = 4 points, I use this technology but not so frequently = 3 points, I am familiar with this technology but do not use it myself = 2 points, I am not familiar with this technology = 1 point |

| **“Digital Care for Mental Health” Attitudes Scale** |
| --- |
| The question prompt was: “How much do you agree or disagree with these statements?” |
| Participants could choose from the following answer choices:  Strongly agree, Agree, Disagree, Strongly disagree, No Opinion* |
| Questions:   1. Monitoring my own behavior (e.g. sleep, physical activity) using a mobile app or wearable device can help support my mental health. 2. Using mobile apps for therapy and learning new knowledge can improve my mental health. 3. Automatically sharing info about my daily activities (e.g. sleep, physical activity) with my care team can improve my mental health. 4. Sharing info about my online activity (e.g. Google searches, Facebook posts) can improve my care. 5. Tracking my own symptoms (e.g. mood, anxiety) using a web or mobile app can help support my mental health. 6. Automatically sharing info about my own symptoms (e.g. mood, anxiety) with my care team can improve my mental health. 7. Ability to communicate with my care team via text messages or mobile app can improve my care. 8. Access to my care team via video conferencing (e.g. Skype, Zoom) can improve my mental health. 9. Feedback via notifications or email about my progress from my care team can improve my mental health. 10. Reminders to follow my team's recommendations (e.g. medications, therapy) can improve my mental health. |
| Notes:  *Scoring: Answer choices were scored as follows: Strongly agree = 5 points, Agree = 4 points, Disagree = 2 points, Strongly disagree= 1 point, No Opinion= 3 points  The question items were grouped into the following categories:   1. Self-help and self-monitoring: items 1, 2, 5, and 10 2. Communication with providers: items 7, 8, and 9 3. Data sharing with treatment team: items 3, 4, and 6 |
