# Supplementary material for: Assessing Health Technology Literacy and Attitudes of Patients in an Urban Outpatient Psychiatry Clinic: Cross-Sectional Survey Study
Source: JMIR Ment Health. 2024 Dec 30;11:e63034. doi: 10.2196/63034 (PMC11729776; doi:10.2196/63034)
Supplement: Multimedia Appendix 2 [file mental_v11i1e63034_app2.docx]

**Table S1.**

|  | | | | | | | | | | | | | | |
| --- | --- | --- | --- | --- | --- | --- | --- | --- | --- | --- | --- | --- | --- | --- |
| A) Mean age comparison between clusters | | | | | | | | | | | | | | |
|  | 1 | | | 2 | |  | | |  | 1 | | | 2 | |
| 2 | **0.02** | | | - | |  |  |  | 2 | **0.03** | | | - | |
| 3 | **<.001** | | | 0.07 | |  |  |  | 3 | **<.001** | | | 0.07 | |
| P-Value Adjustment Method: None | | | | | |  |  |  | P-Value Adjustment Method: FDR | | | | | |
| *Note:* *Table A illustrates the p-values for pairwise comparisons of mean age differences between clusters, assessed using pairwise t-tests with pooled SD. The p-values are shown both without adjustment and with adjustment for multiple comparisons using the False Discovery Rate (FDR) method, with bold numbers indicating statistical significance at the p < .05 level. ‘-’ used to show non-significance.* | | | | | | | | | | | | | | |
| B) Education Level Comparison between Clusters | | | | | | | | | | | | | | |
| Comparison | | 1:2 | 1:3 | | 2:3 | |  | Comparison | | | 1:2 | 1:3 | | 2:3 |
| some hs:hs | | 0.36 | **0.05** | | 1.00 | |  | some hs:hs | | | 0.65 | 0.23 | | 1.00 |
| some hs:some col | | **0.00** | **0.00** | | 0.73 | |  | some hs:some col | | | **0.03** | **0.02** | | 0.94 |
| some hs:col | | **0.01** | **0.00** | | 1.00 | |  | some hs:col | | | 0.07 | **0.02** | | 1.00 |
| some hs:grad | | **0.02** | **0.00** | | 1.00 | |  | some hs:grad | | | 0.11 | **0.01** | | 1.00 |
| hs:some col | | 0.07 | 0.55 | | 0.14 | |  | hs:some col | | | 0.28 | 0.92 | | 0.40 |
| hs:col | | 0.10 | 0.29 | | 0.37 | |  | hs:col | | | 0.34 | 0.63 | | 0.65 |
| hs:grad | | 0.17 | 0.14 | | 0.75 | |  | hs:grad | | | 0.42 | 0.40 | | 0.94 |
| some col:col | | 1.00 | 0.75 | | 0.68 | |  | some col:col | | | 1.00 | 0.94 | | 0.94 |
| some col:grad | | 1.00 | 0.33 | | 0.28 | |  | some col:grad | | | 1.00 | 0.65 | | 0.63 |
| *P* value adjustment method: none | | | | | | |  | *P* value adjustment method: FDR | | | | | | |
| Table B illustrates the *P* values for pairwise comparisons of employment status among clusters, calculated using Fisher exact test. The *P* values are shown both without adjustment and with adjustment for multiple comparisons using the FDR method, with bold numbers indicating statistical significance at the *P*<.05 level.  Abbreviations: some hs= some high school, hs= completed high school, some col = completed some college, col = completed college, grad = some graduate school or completed graduate school. | | | | | | | | | | | | | | |
